# The effect of intensive rehabilitation treatment on sleep disorder in children with motor delays

**DOI:** 10.1186/s12887-023-04067-1

**Published:** 2023-06-15

**Authors:** Sung Hyun Kim, Jin Hee Jung, Min Cheol Chang, Donghwi Park

**Affiliations:** 1 Department of Rehabilitation Medicine, Bundang Jesaeng Hospital, Gyeonggi-Do, Republic of Korea; 2 Department of Rehabilitation Medicine, Yeungnam University Hospital, Daegu, 42415 Republic of Korea; 3 Department of Rehabilitation Medicine, Daegu Fatima Hospital, Ayang-ro 99 gil, Dong-gu,, Daegu, 44033 Republic of Korea

**Keywords:** Sleep problems, Developmental delays, Cerebral palsy

## Abstract

**Background:**

Although the importance of sleep problems has been increasingly emphasized due to the effects on children's development and children's and families' daytime behaviors, physical health, and quality of life, they have been overlooked in clinical practice. However, there have been few studies on the effects of rehabilitation on sleep problems. Therefore, in this study, we investigated the effects of an intensive rehabilitation program on sleep problems in children with developmental delays (DD).

**Methods:**

We included 36 children with DD (30 outpatients, 6 inpatients) and their caregivers who completed all items on the Sleep Disturbance Scale for Children. Of the children with DD, 19 (59.3%) had cerebral palsy (CP) and 13 (40.7%) had DD of non-CP origins, of which 6 (18.8%) had prematurity, 4 (12.5%) had genetic causes, and 3 (9.4%) had an unknown origin. Changes in sleep problems after the intensive rehabilitation program were evaluated using a paired or unpaired t-test, depending on the distribution of the continuous variables.

**Results:**

After the intensive rehabilitation program, in 36 children with DD, there was a significant improvement in the difficulty in initiating and maintaining sleep (DIMS) sub-score (*p* < 0.05). However, there was no significant improvement in the total score or other sub-scores, such as those for sleep breathing disorders (SBD), disorders of arousal (DA), sleep–wake transition disorders (SWTD), disorders of excessive somnolence (DOES), and sleep hyperhidrosis (SH). In the subgroup analysis according to the cause of DD, children with CP had a significant improvement in DIMS and DOES sub-scores (*p* < 0.05).

**Conclusion:**

The intensive rehabilitation program, consisting of more than two sessions per day, effectively alleviated sleep problems in children with DD, especially in those with CP. Among the sleep problems, the intensive rehabilitative program was most effective at improving the DIMS. However, further prospective studies with a larger number of patients with DD and a more standardized protocol are necessary to generalize this effect.

## Background

Although the importance of sleep problems has been increasingly emphasized due to their effects on children's development and children's and families' daytime behaviors, physical health, and quality of life, they have been overlooked in clinical practice. In particular, sleep problems have been more frequently reported in children with developmental delays (DD), such as cerebral palsy (CP) or autism spectrum disorder (ASD), than in children with typical development [1].

Although the prevalence of sleep problems in children with DD has not yet been precisely determined, according to previous studies, the prevalence of sleep problems in children with CP and ASD is 23–46% and 49–89%, respectively, while that in children with typical development is 20–30% [2].

Several studies have explored the causes of sleep disorders in children with DD. In children with CP, sleep disorders may be caused by obstructions due to hypertonia, muscle pain, and reflux [3]. In contrast, sleep initiation difficulties and night waking are common in children with fragile X syndrome, and obstructions due to low muscle tone are common in children with Prader-Willi syndrome [4, 5]. These sleep disorders affect the quality of life of not only children with DD but also their caregivers.

Several medications have been introduced to reduce sleep problems in children [6]. A recent study revealed that administering melatonin significantly impacted the total sleep time and quality of sleep of children with DD [7, 8]. However, despite the few side effects of melatonin, caregivers are reluctant to use sleep-related medicines for children with DD. In addition, selecting medications is difficult because of the different patterns of sleep disorders in clinical practice. Exercise interventions have been shown to improve the sleep of adult patients with neurodegenerative diseases, helping to enhance the sleep quality, reduce the time to fall asleep, and improve the duration of sleep. The beneficial effects on sleep occur through the enhancement of numerous neurotransmitter systems, including those of norepinephrine and serotonin [9, 10].

To date, clinical factors related to the characteristics of sleep problems in children with DD have been reported; however, there have been few studies on the effect of rehabilitation treatments on sleep problems. Therefore, in this study, we investigated the effects of an intensive rehabilitation program on sleep problems in patients with DD.

## Methods

This prospective study was conducted in children with DD who underwent intensive rehabilitation at the Division of Pediatric Rehabilitation of Ulsan Hospital between December 2021 and June 2022. This study was approved by the Institutional Review Board of our hospital (IRB no. 2021–11-003).

### Participants

The study population consisted of children with DD (age 0–14 y; 20 males, 12 females; 32.5% ambulatory) who participated in an intensive rehabilitation program that included physical and occupational treatments and speech, cognitive, and sensory integration treatments, depending on the needs of the children. Children who received intensive care within the last month and those receiving anticonvulsants and spasticity medications were excluded.

The intensive rehabilitation program was composed of one of the following:

1. An admission-based intensive rehabilitation program, consisting of physical and occupational treatment sessions (30 min per session, twice a day, for > 20 times per week for two months) during hospitalization.
2. An outpatient-based intensive rehabilitation program, consisting of physical and occupational treatment sessions (30 min per session, once a day, for > 10 times per week for two months).

### Measurements

The Sleep Disturbance Scale for Children (SDSC) was used to evaluate the degree of sleep disturbance. The SDSC consisted of 26 Likert-type items designed to evaluate specific sleep disorders in children and to provide an overall measure of sleep disturbance suitable for use in clinical screening and research [11]. This scale was previously proven effective in children and adolescents, and recently, it was also proven in infants and toddlers, aged 6–36 months [12, 13].

The SDSC has a total sleep score and the following six subscales: sleep-breathing disorders (SBD), disorders of excessive somnolence (DOES), difficulty in initiating and maintaining sleep (DIMS), sleep–wake transition disorders (SWTD), disorders of arousal (DA), and sleep hyperhidrosis (SH). Question 1 was ‘How many hours of sleep does your child get on most nights’, with responses ranging from 1 (‘9–11 h’) to 5 (‘less than 5 h’). Question 2 was ‘How long after going to bed does your child usually fall asleep’, with responses ranging from 1 (‘less than 15’) to 5 (‘more than 60’). The scale for responses to the remaining questions, for example, Question 13, ‘the child has difficulty in breathing during the night’, ranged from 1 (‘Never’) to 5 [‘Always (daily)’]. Scores are tallied to provide a total sleep score as well as a score for each of the six sleep disorder subscales: SBD (three items), DOES (five items), DIMS (seven items), SWTD (six items), DA (three items), and SH (night sweats; two items).

Raw scores were converted to t-scores, and a T-score > 70 (> 95th percentile) indicated a clinically significant sleep problem. The SDSC was developed using a sample of typically developing children (*N* = 1,157) and children with sleep disorders (*N* = 147), aged 6.5 to 15.3 years and was designed to group sleep disorders frequently reported in children. A previous study reported an acceptable sensitivity (89%) and specificity (74%) in distinguishing between the control group and children with sleep disorders [14].

The SDSC was completed by the caregivers before the start of intensive rehabilitation; the same SDSC was completed again within a month of completing 2 months of intensive rehabilitation.

### Sample size calculation

The SDSC score was obtained from a pilot sample of 10 children, and it was used to perform a two-tailed sample size calculation for the expected mean differences, with a standard deviation of 0.2, a level of significance of 0.05, and a power of 80%. Accordingly, the required sample size for this study was at least 13 patients in each group [15].

### Statistical analysis

Statistical analyses were performed using SPSS for Windows and the R package for Windows (version 2.15.2, R Foundation for Statistical Computing, Vienna, Austria). To evaluate improvements in sleep problems after the intensive rehabilitation program, *p*-values were calculated using a paired or unpaired t-test, depending on the distribution of the continuous variables. The results are presented as mean ± standard deviation. Statistical significance was set at *P* < 0.05.

## Results

### Patient characteristics

Thirty-two children with DD were enrolled. Among them, 20 were male and 12 were female (mean age, 4.19 ± 3.27 years). The demographic data of patients with DD are shown in Table 1. A total of 32 patients and their caregivers agreed to participate in the study. The SDSC was completed by caregivers who tended the children while they slept. The SDSC was distributed in person, and 30 caregivers (83.3%) returned for the follow-up SDSC. One patient took spasticity medication, and two patients took anticonvulsant medications.

**Table 1:** Classification of the causes of DD is presented

	N	%
CP	19	59.4
Prematurity	6	18.8
Genetic	4	12.5
Other	3	9.4
total	32	100

*DD* developmental delay, *CP* cerebral palsy, *N* number of patients

Of the children with DD, 19 (59.3%) had CP, and 13 (40.7%) did not have CP. Compared to children with DD who did not have CP, those with CP had lower Gross Motor Function Measures (GMFM) and higher total SDSC scores, but the differences were not significant (Table 2). The average GMFM was 51.98 in CP and 56.73 in non CP, indicating a lower level of GMFM in CP. After intensive rehabilitation treatment, GMFM was observed in all children, and among them, the non CP showed more improvement with 64.69 (Table 2). Among non CP, there were 6 (18.8%) with prematurity, 4 (12.5%) children with genetic causes, and 3 (9.4%) with an unknown origin (Table 1). Among prematurity, children with brain injury and spasticity was diagnosed with CP, and those with only developmental delay without brain lesion or spasticity were diagnosed with prematurity.

**Table 2:** Demographic and clinical characteristics of children with developmental disorder (*N* = 32)

	Sex (n)		Age (year)	Follow up interval(day)	GMFM(%)	SDSC (T-score)^a^						
	**Male**	**Female**				**DIMS**	**SBD**	**DA**	**SWTD**	**DOES**	**SHY**	**Total**
CP (*N* = 19)	12	7	5.35 ± 3.48	77.95 ± 81.67	46.33 ± 22.74	61.47 ± 14.38	52.63 ± 7.97	50.00 ± 7.48	58.42 ± 8.28	58.37 ± 16.22	49.89 ± 7.32	60.53 ± 13.34
Non-CP (*N* = 13)	10	3	2.48 ± 2.17	59.62 ± 30.81	60.12 ± 20.69	60.69 ± 9.85	56.85 ± 9142	47.85 ± 3.05	61.92 ± 9.64	45.62 ± 4.31	53.69 ± 8.64	57.92 ± 9.96
Total (*N* = 32)	22	10	4.19 ± 3.27	70.50 ± 65.75	54.75 ± 21.92	61.16 ± 12.56	54.34 ± 8.70	49.13 ± 6.11	59.84 ± 8.88	53.19 ± 14.16	51.44 ± 7.97	59.47 ± 11.98

Values are mean ± standard deviation (%)

*CP* cerebral palsy, *GMFM* Gross Motor Function Measures, *SDSC* Sleep Disturbance Scale for Children, *DIMS* Disorders of Initiating and Maintaining Sleep, *SBD* Sleep Breathing Disorders, *DA* Disorders of Arousal, *SWTD* Sleep–Wake Transition Disorders, *DOES* Disorders Of Excessive Somnolence, *SHY* Sleep Hyperhydrosis

^a^Score based on T-score

A total of 32 children with DD received intensive rehabilitation, and their caregivers chose the program. Of these, 28 and 4 children underwent outpatient- and admission-based intensive rehabilitation programs, respectively.

### Sleep problem patterns

Children with DD had the following mean T-scores: DIMS, 61.16 ± 12.56 (min–max: 41–100); SBD, 54.34 ± 8.70 (min–max: 45–79); DA, 49.13 ± 6.11 (min–max: 47–70); SWTD, 59.84 ± 8.88 (min–max: 41–95); DOES, 53.19 ± 14.16 (min–max: 42–100); SHY, 51.44 ± 7.97 (min–max: 45–93); and total score, 59.47 ± 11.98 (min–max: 41–100) (Table 1). Children with CP had the following mean scores: DIMS, 61.47 ± 14.38 (min–max: 41–100); SBD, 52.63 ± 7.97 (min–max: 45–79); DA, 50.00 ± 7.48 (min–max: 47–70); SWTD, 58.42 ± 8.28 (min–max: 41–73); DOES, 58.37 ± 16.22 (min–max: 42–95); and SHY, 49.89 ± 7.32 (min–max: 45–69). Children without CP had the following mean scores: DIMS, 60.69 ± 9.85 (min–max: 45–76); SBD, 56.85 ± 9.42 (min–max: 45–72); DA, 47.85 ± 3.05 (min–max: 47–58); SWTD, 61.92 ± 9.64 (min–max: 50–75); DOES, 45.62 ± 4.31 (min–max: 42–53); and SHY, 53.69 ± 8.64 (min–max: 45–69) (Table 2).

### The effect of the intensive rehabilitation program on sleep problems

After the intensive rehabilitation program, there was a significant improvement in the DIMS sub-score (*P* < 0.05) (Table 3). However, there was no significant improvement in the total score or in the other sub-scores, such as those for SBD, DA, SWTD, DOES, and SHY. In the subgroup analysis according to the cause of DD in children with CP, there were significant improvements in the DIMS and DOES sub-scores (*P* < 0.05). However, in the CP group, there was no significant improvement in either the sub-scores or total scores (*P* ≥ 0.05). There was no significant difference of SDSC between admission and out-patient based intensive rehabilitation program (*P* ≥ 0.05).

**Table 3:** The degree of change in sleep disturbance scale for children after intensive rehabilitation program

SDSC (T-Score)^a^							
	ΔDIMS	ΔSBD	ΔDA	ΔSWTD	ΔDOES	ΔSHY	ΔTotal
CP	-4.68 ± 10.39	0.79 ± 8.26	-0.05 ± 9.32	3.11 ± 12.00	-3.33 ± 8.59	1.47 ± 9.60	-2.32 ± 12.84
*p*-value	0.038*	0.682	0.981	0.274	0.038*	0.183	0.411
Non-CP	-2.85 ± 8.89	-2.92 ± 6.78	1.69 ± 4.13	-2.54 ± 8.80	7.00 ± 11.87	-4.15 ± 8.60	-0.23 ± 7.98
*p*-value	0.271	0.146	0.165	0.319	0.055	0.107	0.919
Total	-3.94 ± 9.70	-0.72 ± 7.80	0.66 ± 7.61	0.81 ± 11.02	1.46 ± 11.34	-0.81 ± 9.49	-1.47 ± 11.02
*p*-value	0.029*	0.606	0.629	0.68	0.85	0.954	0.428

Values are mean ± standard deviation (%)

*CP* cerebral palsy, *SDSC* Sleep Disturbance Scale for Children, *DIMS* Disorders of Initiating and Maintaining Sleep, *SBD* Sleep Breathing Disorders, *DA* Disorders of Arousal, *SWTD* Sleep–Wake Transition Disorders, *DOES* Disorders Of Excessive Somnolence, *SHY* Sleep Hyperhydrosis

^a^Score based on T-score

* p-value <0.05

## Discussion

Most previous studies of the correlation between physical therapy and sleep problems included adult patients with neurological disorders, such as stroke, Parkinson’s disease, and multiple sclerosis [16–18]. In those studies, physical therapy programs tended to have positive effects on sleep problems, although it is difficult to generalize the results due to the relatively few studies and heterogeneity of the interventions. Specifically, physical therapy programs that stimulate aerobic metabolism and muscle endurance seem to be useful in improving sleep efficiency and quality objectively measured using polysomnography. In a previous study of the mechanism of sleep problem improvements after exercise programs, sleep-related biomarkers, such as melatonin, serotonin, and cortisol, were evaluated [18]. Although the study focused on patients with multiple sclerosis, exercise training of moderate intensity led to an increase in serotonin values [18]. Moreover, there was a significant correlation between the increase in serotonin levels and improvements in the quality of sleep after 6 weeks of physical training [18]. Thus, in our study, improvements in sleep problems after intensive rehabilitation treatment, especially in children with CP, are thought to be associated with improvements in aerobic metabolism and muscle endurance or increases in serotonin levels. Further studies are necessary to confirm this hypothesis.

Among the sleep problems of the children in our study, DIMS was the most severe, followed by SWTD, SHY, DOES, SBD, and DA. Among children with CP, DIMS was the most severe, followed by SWTD, DOES, DA, SBD, and SHY. In patients with conditions other than CP, SWTD was the most severe, followed by DIMS, SBD, SHY, DA, and DOES. In previous studies, the most frequently occurring pediatric sleep disorders among all children were difficulties initiating or maintaining sleep and sleep-disordered breathing [19]. Difficulties initiating or maintaining sleep affect 40% to 75% of children with developmental delays—two to three times the rate among typically developing children); however, daytime fatigue (33.3%) and difficulty falling sleep (30%) were the most frequently reported sleep problems occurring often or always in children with CP [20–22]. The characteristics of these sleep problems in our study are in agreement with those in previous studies [20–22], suggesting that sleep problems occur in a noticeable proportion of patients with DD and CP.

Previous studies introduced treatment options for sleep problems, such as behavioral treatments or medications [23–25]. However, there have been few studies on the effect of intensive physical therapy on sleep problems in children with DD. The results of our study showed that intensive rehabilitation programs were effective in improving sleep patterns, especially in DIMS, which is one of the most problematic sleep problems in children with DD, especially in children with CP. Considering the parents' resistance to starting medication and the difficulty of taking drugs for children with DD, our study showed the possibility of using an intensive rehabilitation program as a treatment method for sleep disorders in children with DD.

This study had a few limitations. First, there was a relatively small number of DD patients and limited heterogeneity of DD causes. To generalize the effect of intensive rehabilitation programs, further studies with a larger number of patients with DD, including each cause of DD, are necessary. Second, our study did not include a uniform rehabilitation treatment program. However, in the previous study, more than 5 times of treatment sessions a week was defined as high frequency treatment, and it was proved that high frequency treatment showed a difference in effects on development scores compared to low frequency treatment, which is defined as less than 5 times of treatment sessions a week, in children with CP under 36 months [26]. Therefore, we defined the same as an intensive rehabilitation program, although the number of treatment sessions for admission and out patient based intensive treatment was different. However, further prospective studies with standardized rehabilitation treatment programs are necessary to generalize the results of intensive rehabilitation programs.

## Conclusion

An intensive rehabilitation program consisting of over two sessions per day is effective in alleviating sleep problems in children with DD, especially in children with CP. Among the sleep problems, the intensive rehabilitation program was most effective in terms of addressing difficulties in initiating and maintaining sleep. However, further prospective studies with a larger number of patients with DD and a more standardized protocol are necessary to generalize this effect.

## Data Availability

The datasets used and/or analysed during the current study available from the corresponding author on reasonable request.

## Abbreviations

DD: Developmental disorder
DIMS: Difficulty in initiating and maintaining sleep
CP: Cerebral palsy
DOES: Disorders of excessive somnolence
ASD: Autism spectrum disorder
SDSC: Sleep Disturbance Scale for Children
SBD: Sleep-breathing disorders
SWTD: Sleep–wake transition disorders
DA: Disorders of arousal
SH: Sleep hyperhidrosis
